# SLAM algorithm applied to robotics assistance for navigation in unknown environments

**DOI:** 10.1186/1743-0003-7-10

**Published:** 2010-02-17

**Authors:** Fernando A Auat Cheein, Natalia Lopez, Carlos M Soria, Fernando A di Sciascio, Fernando Lobo Pereira, Ricardo Carelli

**Affiliations:** 1 Institute of Automatics, National University of San Juan, Argentina; 2 Medical Technology Department, National University of San Juan, Argetnina; 3 Department of Electrical and Computer Engineering, Faculdade de Engenharia da Universidade do Porto, Portugal

## Abstract

**Background:**

The combination of robotic tools with assistance technology determines a slightly explored area of applications and advantages for disability or elder people in their daily tasks. Autonomous motorized wheelchair navigation inside an environment, behaviour based control of orthopaedic arms or user's preference learning from a friendly interface are some examples of this new field. In this paper, a Simultaneous Localization and Mapping (SLAM) algorithm is implemented to allow the environmental learning by a mobile robot while its navigation is governed by electromyographic signals. The entire system is part autonomous and part user-decision dependent (semi-autonomous). The environmental learning executed by the SLAM algorithm and the low level behaviour-based reactions of the mobile robot are robotic autonomous tasks, whereas the mobile robot navigation inside an environment is commanded by a Muscle-Computer Interface (MCI).

**Methods:**

In this paper, a sequential Extended Kalman Filter (EKF) feature-based SLAM algorithm is implemented. The features correspond to lines and corners -concave and convex- of the environment. From the SLAM architecture, a global metric map of the environment is derived. The electromyographic signals that command the robot's movements can be adapted to the patient's disabilities. For mobile robot navigation purposes, five commands were obtained from the MCI: turn to the left, turn to the right, stop, start and exit. A kinematic controller to control the mobile robot was implemented. A low level behavior strategy was also implemented to avoid robot's collisions with the environment and moving agents.

**Results:**

The entire system was tested in a population of seven volunteers: three elder, two below-elbow amputees and two young normally limbed patients. The experiments were performed within a closed low dynamic environment. Subjects took an average time of 35 minutes to navigate the environment and to learn how to use the MCI. The SLAM results have shown a consistent reconstruction of the environment. The obtained map was stored inside the Muscle-Computer Interface.

**Conclusions:**

The integration of a highly demanding processing algorithm (SLAM) with a MCI and the communication between both in real time have shown to be consistent and successful. The metric map generated by the mobile robot would allow possible future autonomous navigation without direct control of the user, whose function could be relegated to choose robot destinations. Also, the mobile robot shares the same kinematic model of a motorized wheelchair. This advantage can be exploited for wheelchair autonomous navigation.

## Background

Integration of robotic issues into the medical field has become of a great interest in the scientific community in the recent years. Mechanical devices specially developed for surgery like robot manipulators, control algorithms for tele-operation of those robots and cognitive algorithms for user decision learning are some examples of robotic applications in medicine. On the other hand, service, assistance, rehabilitation and surgery can also benefit from advances in robotics.

The objective of SLAM is to concurrently build a map of an environment and allow the robot to localize itself within that environment [1-3]. Although SLAM is not only devoted to mobile robots [4], it was first thought as a tool for mobile robot autonomous navigation [5-7]. Since its early beginnings [8,9], the SLAM scheme has been developed and optimized in different ways. The most common implementation uses an Extended Kalman Filter (EKF) [6,7]. The EKF minimizes the mean quadratic error of the system state and considers all variables as Gaussian random variables [5,9]. The map obtained by an EKF-based SLAM implementation is usually a feature-based map [10-12]. The features of the map obey some geometrical constrain of the environment [13,14]. Thus, in [14] is presented a line-based SLAM where lines are related to walls; in [12] is shown a point-based SLAM where all significant points are related to trees of the environment. Other approaches use a Particle Filter, [15,16], for solving the SLAM problem. The Particle Filter SLAM implementation has the advantage that the features of the map are not restricted to be Gaussian. In [15], there is a SLAM approach based on the Unscented Kalman Filter which gives a better performance of the SLAM scheme considering the non-linearity of the model of the vehicle and the model of the features. The best SLAM algorithm for a particular environment depends on hardware restrictions, the size of the map to be built by the robot and the optimization criterion of the processing time. A common criterion of all SLAM algorithms is that they must be consistent [17,18]. A non consistent SLAM derives in unreliable environmental and position information.

The integration of the SLAM algorithm with mobile robot navigation strategies are discussed in [19]. Thus, in [19] it is shown the implementation of the SLAM algorithm to autonomous navigation. Autonomous navigation in SLAM requires that the robot is able to decide by its own the destination within the environment being mapped. On the other hand, in [20] is shown an implementation of a semi-autonomous navigation strategy in SLAM. In this application, the mobile robot has some knowledge about its state but it cannot take any action according to that.

The use of biological signals to command robotic devices has been widely discussed in the scientific literature. Thus, Brain-Computer Interfaces developments to govern the motion of a mobile robot can be found in [21]. Also, face posture recognition and electromyographic signals were used to command robotic devices [22]. As an example of these implementations, in [23] is shown a Muscle-Computer Interface to direct the motion of a robot manipulator, simulating an orthopaedic arm. Most of these biological signals interfaces are joystick based devices. The biological signal is acquired, filtered, classified and then a feature or a pattern is extracted from it. According to the extraction results, the pattern or feature has a command -or an action- associated with it. Thus, Finite State Machines have been proposed as actuator devices of the biological signals interfaces, [21,23].

In this work we focus the human-machine interface in the EMG signals, which has been extensively used for wheelchair navigation, as in [24-26]. In the former, the operator controls the direction of motion by means of EMG signal from the neck and the arm muscles. An intuitive human-machine interface is implemented by mapping the degrees of freedom of the wheelchair onto the degrees of freedom of the neck and arm of the operator. For disabled users (C4 or C5 level spinal cord injury), an EMG based human-computer interface (HCI) is proposed in [25], where the user expresses his/her intention as shoulder elevation gestures and their combination defines the command control of the wheelchair.

When the operator preserves the capability to control a joystick to command a powered wheelchair, it becomes as a better solution for the wheelchair navigation. Thus, the SLAM algorithm would not be necessary since the patient has a complete maneuverability over the wheelchair and map information is not longer needed. On the other hand, the EMG control is an alternative for disabled people who cannot use a traditional joystick (i.e. amputees, persons with progressive neuromuscular disorders, and so on). The MCI can be adapted to any pair of agonist-antagonist muscles, like facial, shoulder or neck muscles and not only for extremities. In the first stage of amyotrophic lateral sclerosis (ALS) some muscles remain actives, even in the upper extremities. In others neuromuscular disorders such as Duchenne muscular dystrophy (DMD), low level spinal cord injury (B at Spinal Cord Injury Classification System) and Brown-Séquard syndrome (between some pathologies) the remaining capabilities of the user can be used as command control of any robotic device.

For physically disabled or aged people, the intelligent wheelchairs allow them to achieve some degree of independent personal mobility with little or no external aid. An example of intelligent wheelchairs is the robotic wheelchair developed by the Federal University of Espirito Santo, Brazil, which has the capability of navigating within a sensorized equipped environment [27].

Combining robotics techniques with Human-Machine Interfaces has led to a slightly explored research area. Mobile vehicles equipped with sensor systems or manipulators are developed to offer autonomous or semi-autonomous navigation, suitable for severely disabled users. The MOVAID, [28], project and the Nursery Robot System for the elderly and the disabled [29] are examples of the robotic incursions in assistance tasks.

An interesting application of these systems is transportation in hospitals, such as delivery of meals, pharmacy supplies and patient records, or to hold and carry an adult patient or a disabled child. Different navigation algorithms were implemented in the HelpMate robot [30] -which requires a partially structured environment- and the MELKONG care robot controlled by a nurse or by a patient by means of a joystick, [31].

The use of a mobile robot or a robotic wheelchair as a transportation system for a person with motor disabilities provides him/her with some degree of autonomy subordinated to the sensorized environmental configuration combined with the reactive behavior features of the vehicle, and with the patient's capability to control the entire system. In order to achieve complete autonomy, the user of the wheelchair should be able to navigate in the environment where she/he stands without the assistance of extra people. To do so, the user should have some level of knowledge of the environment. When it is a sensorized one, the patient is restricted to that place and any intention to go out of that environment will violate the autonomy. In this case, a map construction and robot localization appears as an appropriate solution for reaching autonomy, which is the objective of the present work. The central contribution of this paper is the combination of an MCI with an SLAM algorithm for the navigation of a robotic wheelchair within unknown and unsensorized environments.

In this work, a mobile robot controlled by a Muscle-Computer Interface is presented. Although the MCI used can be adapted to the patient capabilities, in this work, flexion, extension, hand pronation and hand supination of the right arm were used. The robot is equipped with a sequential EKF-based SLAM algorithm to map the unknown environment and with low level behavioral strategy to avoid collisions. Once the patient activates the SLAM algorithm, a map of the environment is continuously acquired. This map is a feature-based map with metric information of the environment. The features extracted correspond to corners (convex and concave) and lines (representing walls). A secondary map is also developed with the information of the segments associated with each line of the environment. This parallel map is corrected and updated according to the SLAM system state and the evolution of the covariance system matrix of the SLAM algorithm. Once the SLAM is turned off by the patient, the built map is stored in a computer system. The SLAM process allows dynamic and semi-structured indoor environments. A kinematic control law to command the motion of the robot was also implemented.

The system proposed in this paper allows the construction of geometric maps of unknown environments by a vehicle governed by a MCI. The obtained map is then stored and it can be used for future safe navigation purposes. The map remains stored on the computational system and if the user of the MCI positions the vehicle within a previously mapped environment he/she can use the map information for planning tasks. The system presented here can be directly applied to intelligent wheelchairs without the need of using previously known environments. The SLAM algorithm avoids the use of off-board sensors to track the vehicle within an environment -a sensorized environment restricts the area of movements of an intelligent wheelchair to the sensorized area-. Furthermore, the SLAM algorithm allows the creation of maps of unvisited regions.

## Methods

### System architecture

**Figure 1:**
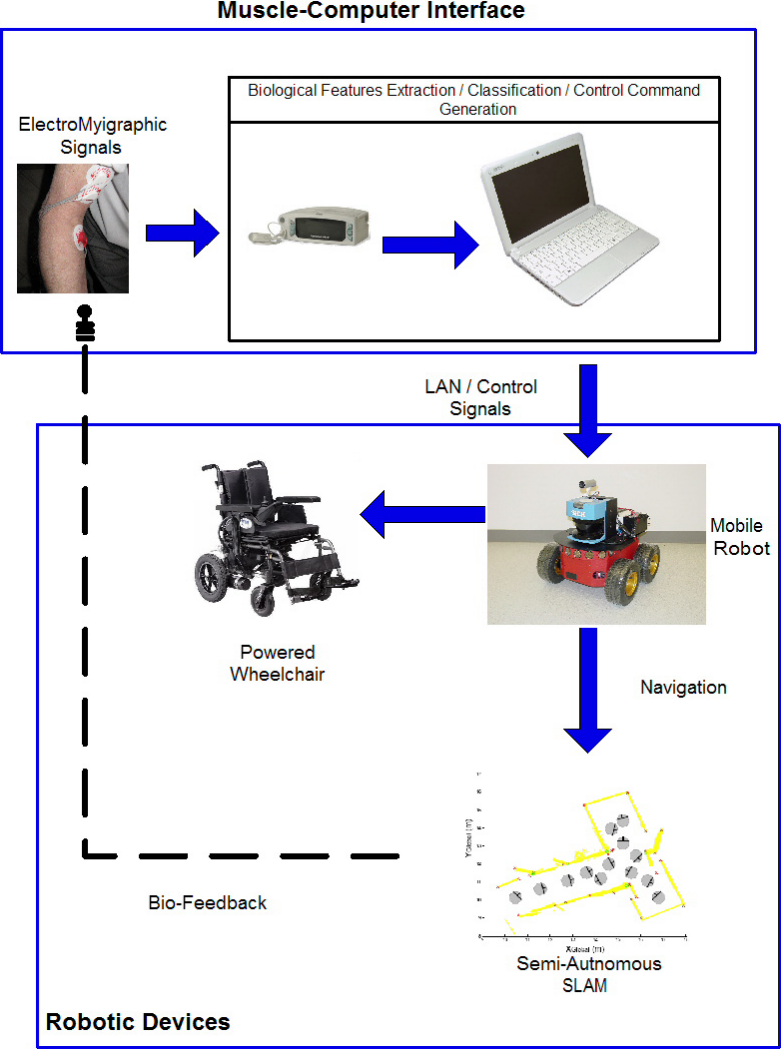
**General system architecture**. It is composed by two main sub-systems. One acquisition and processing of the biological signals and a second sub-system for the robot motion control and intelligence.

Figure 1 shows the architecture of the implemented system. This architecture has two main sub-systems: a first system managing the biological signal processing and a second system that manages the robotic devices involved in the entire process.

In figure 1, the Muscle-Computer Interface extracts and classifies the surface electromyographic signals (EMG) from the arm of the volunteer. From this classification, a control vector is obtained and it is sent to the mobile robot via Wi-Fi.

The Robotic Devices sub-system is composed by the SLAM algorithm, the map visualization and managing techniques, the low level robot controllers and the bio-feedback interface to ensure the system's stability. If no bio-feedback is presented to the patient, the system becomes open loop and the robot could collide [21].

### Electromyographic signals extraction and classification

After previous informed consent, EMG data corresponding to four classes of motion were collected from 7 volunteers with normal cognitive capabilities: two below-elbow amputees, three elder and two young normally limbed patients using a pair of bipolar Ag/AgCl (3 M RedDot) electrodes according to SENIAM [32] protocol. The electrodes were located at the *biceps brachii*, *triceps brachii*, *supinator *and *pronator teres *muscles and the reference electrode was placed on the right ankle. Electronic amplification, isolation, and filtering are implemented by a front-end signal conditioning circuit with the following characteristics:

Input impedance: 10 MΩ

Gain: 1000

Common Mode Rejection Ratio (CMRR): 120 dB

Band-Pass Filter: 10-500 Hz.

Thereafter, the analogue EMG signals were digitized at a sampling rate of 1000 Hz with an A/D 6015 board (National Instruments^®^).

In the initial setting stage, the system records the maximum voluntary contraction (MVC) and the background noise. Afterwards, with this information, the system executes an adaptation routine for the current environmental conditions and for the current specific characteristics of each volunteer. The volunteers were prompted to perform the following types of contraction: elbow flexion/extension, wrist pronation/supination (elbow rotation for amputees) and no movement (rest). With these results, a simple classifier was implemented, in order to generate a set of commands based in muscle contractions. For navigation purposes, the natural choice is to command the robot with the pronation and supination from the right hand (or left, according to the volunteer's decision). These EMG signals were recorded only in *biceps *and *pronator *muscles, in order to prevent the interchannel crosstalk. The other acquisition channels are not taken into account for control purposes.

### Signal control generation

The control vector generated by the EMG signals is a set of basic commands to allow the robot's movements over the environment. These commands are described in Table 1. To accomplish the set of commands shown in Table 1, the following considerations were taken into account.

**Table 1:** Commands for navigational purposes generated by the user

Generated Commands	Description
Start	SLAM and control algorithms begin
Stop	Stops robot's movements but the SLAM algorithm continues
Turn to the left	Sustained command to make the robot turning to user's left
Turn to the right	Sustained command to make the robot turning to user's right
Exit	SLAM stops and a map is visualized

i. The linear velocity of the robot is a function of its angular velocity control input. If no angular velocity is present, then the linear velocity remains constant.

ii. The *Start*, *Stop *and *Exit *signals are accomplished by the extension of the forearm. It is so because this signal is present only in the triceps and no other muscle can activate it. Thus, a first extension is needed to start the SLAM algorithm. A second extension would stop it. If two extensions with elapsed time between them lower than 5 seconds are presented, then the SLAM algorithm is turned off.

iii. Turn to the left command is related to the pronation movement.

iv. Turn to the right is related to the supination movement.

v. The EMG signal is full-wave rectified and a sixth order Butterworth low-pass filter (cutoff frequency: 10 Hz) is implemented in order to smooth the trace and extract the envelope.

**Figure 2:**
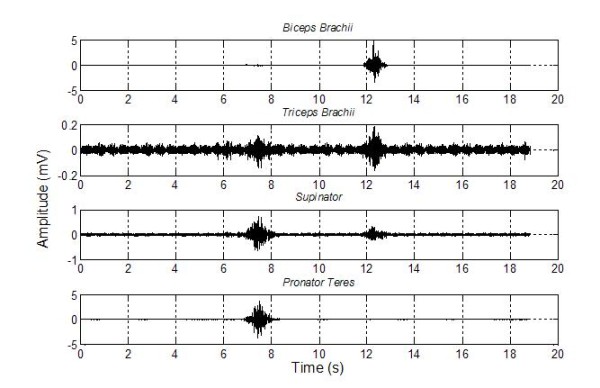
**Electromyographic signal acquisition I**. Four channels of electromyographic signal acquisition. In the *Triceps Brachii *channel the crosstalk amplitude has the same order of the baseline.

In figure 2 different signals of the MCI are shown according with the four acquisition channels. As a first approach the angular velocity of the mobile robot, related to pronation (turn to left) and supination (turn to right) is assigned to the signal of *pronator teres *and *biceps brachii *muscles. The classification was implemented through the difference between channels, in order to minimize the interchannel crosstalk. The positive values of this signal are related to the positive angular velocity provided to the robot (the robot turns to the left). The negative values correspond to negative angular velocity (the robot turns to the right).

Since the EMG can be considered as a zero-mean stochastic process, amplitude appears as proportional to the standard deviation (STD) varying in time. Under this assumption, Mean Absolute Value (MAV) is proposed as maximum likelihood estimator of the EMG amplitude (and, consequently, EMG-muscle force relation) [33]. MAV is defined as in Zecca *et al*. [34],

where *emg*(*j*) stands for the *j-th *sample from the beginning of the experiment and *k *is the current sample. This equation was modified to be applied in a recursive way, that is, more suitable for real-time control,

where *k *= 1,2,... corresponds to the sample time and *emg*(*k*) is the myoelectric signal in each sampled time.

During the test, the subject was instructed to use a pair of agonist-antagonist muscles (i.e. *pronator teres *and *biceps brachii *muscles) to command the robot. The muscle contraction amplitude is estimated through MAV, which, in turn, is transformed to angular velocity of the mobile robot. The decision criterion of the direction of turning is the sign obtained from the difference between MAV signals, that is, *sign*(*emg*_*channel*1_(*k*))- *emg*_*channel*2_(*k*))

### Robotic devices

**Figure 3:**
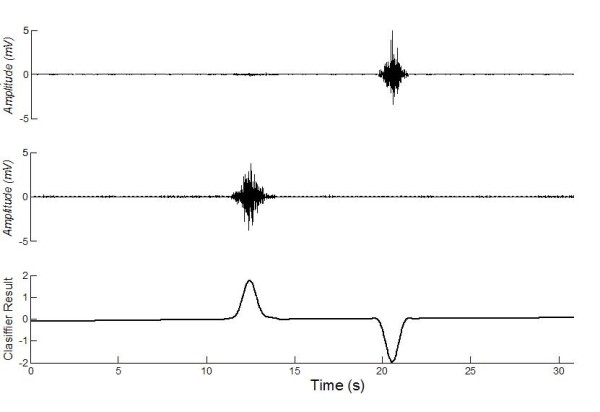
**Electromyographic signal acquisition II**. *Biceps B*. contraction and *Pronator T *contraction are shown in the top and middle panels respectively. The output of the classifier is shown in the bottom panel (*S*_*control*_).

Once obtained the *S*_*control *_(output classifier) from the classification stage -as shown in figure 3-, it is applied into the robot. The robot used in this work, is a non-holonomic of the unicycle type Pioneer 3AT built by ActivMedia^® ^-shown in figure 1. The kinematic equations of this robot are shown in (1).(1)

In (1), *V(k) *and *W(k) *are the linear and angular velocities, respectively; *x(k)*, *y(k) *and *θ(k) *are the current pose of the robot -*x(k) *and *y(k) *represent the robot position and *θ(k) *its orientation in a global coordinate system- and Ω_*x*_*(k)*, Ω_*y*_*(k) *and *Ωθ(k) *are the additive uncertainties associated with the robot's pose at the time instant *k*. Given that the mobile robot used here has the same kinematic model (1) than a powered wheelchair, the results shown in this section could be directly applied to the wheelchair [34] -without consideration of dynamic variables-.

**Figure 4:**
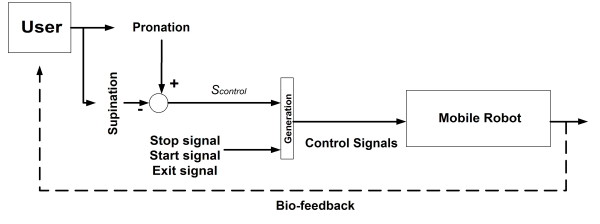
**Mobile robot control system**. Mobile robot control system.

The control signals that are provided to the robot, *V(k) *and *W(k)*, are generated by injecting *S*_*control *_into the mobile robot (as shown in figure 4). Thus, if it is positive, the robot turns to the left, and, if negative, turns to the right. The control law implemented is shown in (2).(2)

In (2), *V*_*max *_and *W*_*max *_are, respectively, the maximum linear and angular velocities allowed by the MCI. Also, *S*_*control *_is the control signal; Δ*t *is the sampled time of the time-discretized system. As it is shown in (2), the linear velocity is incremented when the angular velocity decreases and vice versa. This means that when the robot is turning, its linear velocity decreases. If no angular velocity is present, then the robot travels with a constant maximum linear velocity. Figure 4 shows a scheme of the control system.

In figure 4, the *Stop*, *Start *and *Exit *signals along with *S*_*control *_are transmitted to the robot via a Wi-Fi connection.

Also, the mobile robot is equipped with a laser sensor (built by SICK^®^) which acquires 181 range measurements of the environments in 180 degrees. The maximum range of the sensor is of 30 m.

### SLAM algorithm

The SLAM algorithm implemented in this work is a sequential EKF-based SLAM (EKF, Extended Kalman Filter). The EKF-SLAM of this paper is a feature extraction based algorithm that uses corners (convex and concave) and lines of the environment as features to localize the robot and, simultaneously, to build the map, [29]. All extracted features and the robot's pose are located at a global coordinates system. In parallel with the map built by the SLAM algorithm, a second map of the segments associated to line features is maintained. This secondary map stores the information concerning the beginning and ending points of each segment. Due to the fact that lines of the environment are associated with walls, the parameters of a single line can represent two o more walls on the real environment -e.g., two walls on both sides of a door-. A map built by lines has no information for navigation purposes because all walls belonging to one line are going to be considered as a single feature. This problem is solved by using the secondary map of segments, which gives a metric tool to differentiate line features.

The map of an environment is the set of features that represent it. In the SLAM, the *robot-map *system is interpreted as one single vector state. Thus, at instant *k *the robot pose is defined as *x*_*v*_(*k*) and the *i*^*th*^*-feature *is expressed as *p*_*i*_(*k*). The *p*_*i*_-vector contains the parameters that define the *i*^*th*^*-feature*. The complete state of the system, with *n *features, is(3)

The mean and covariance of the state shown in (3) are, respectively,(4)

Here, Σ_*vv*_(*k*|*k*) is the covariance matrix of the robot pose estimate at instant *k*, Σ_*ii*_(*k*|*k*) is the *i*^*th*^*-feature *covariance estimate, and Σ_*jj*_(*k*|*k*) is the cross covariance between the *i*^*th*^-element and the *j*^*th*^-element of the vector state (4). It is assumed that all features in (3) are stationary and without process noise. According to this, the process model can be expressed as(6)

In (6), *u*(*k*) is the control input previously defined in (2) and Ω(*k*) is the mobile robot process noise.

The observation model is(7)

In (7), the observation model is a function of the robot pose (*x*_*v*_), the environmental features (*p*_*i*_) and the noise associated to the sensor -*ψ*- (range laser built by SICK^®^). No odometric encoder information was used in the SLAM algorithm implemented in this work. Given that (1) and (7) are non-linear equations, the SLAM algorithm requires the linearization of such expressions [35].

### Extended Kalman filter

In order to accomplish the estimation of the vector state shown in (3), an EKF filter is used. The implementation of an EKF-based SLAM algorithm is widely presented in the literature ([6,7]). In this work, a sequential EKF-SLAM was implemented to reduce the processing time involved in the SLAM execution loop.

### Feature extraction

The lines are defined by two parameters: the distance (*ρ*) of the line to the global coordinate center frame and the angle (*α*) between the global *x-axis *and a vector, normal to the detected line, as shown in [14]. The points measured by the sensor along a line, are determined by an iterative clustering algorithm. The set of points that actually belong to a line are processed by a linear regression algorithm in order to calculate the best line [14].

Considering that a line, by hypothesis, is a Gaussian random variable whose parameters, (*ρ*, *α*), are considered as the mean values. The covariance matrix for a detected line is obtained by Taylor series propagation of the covariance of the regression. As a line is detected, an array with information about the segment associated with that line is created. The secondary map of the SLAM, stores both endpoints of all segments in a global coordinates frame. The secondary map is updated and corrected according to the EKF map improvement. Therefore, if a new line is added to the map, both its segment endpoints are also added to the secondary map.

Although lines intersection could be used for corners extraction, an independent method is used in this paper. By using an independent method for feature detection, possible cross-correlations between features parameters are avoided. The detection of convex and concave corners of the environment is made by applying a right and left differentiation on the actual angle provided by a range sensor measurement. Those points, for which the derivate of the angle exhibits a discontinuity along the direction, are chosen as possible corners. Then, by clustering the neighborhood of each chosen point and analyzing the metric relations between points in that cluster, it is possible to find the estimated corners.

**Figure 5:**
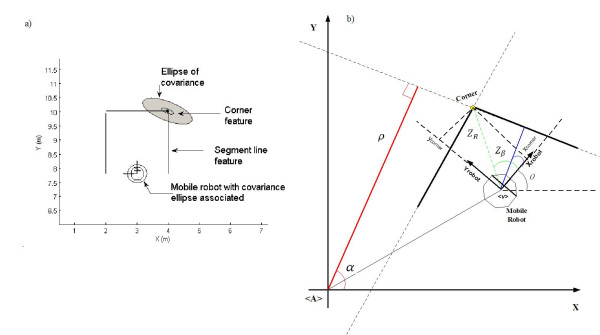
**Features detection of the environment**. Line segment and corner detection by a mobile robot. a) The robot and the detected corner show the covariance ellipse associated to them. b) Detection of line and a corner. Both features, the lines and corners, represent the environment through which the mobile robot navigates. The parameters of both features correspond to the ones shown in Eqs. (8) and (9): *ρ *is the distance of the line to the origin of the coordinate system and *α *the angle between the *x*--axis and a normal to that line; on the other hand, *Z*_*R *_is the distance of the robot to the corner and *Z*_*β *_is the angle between the *x*--axis of the robot and the corner.

Finally, the detected corner is also treated as a Gaussian random variable and its parameters are represented in the global reference frame --. Figure 5a shows an example of the corners detection algorithm in a closed environment. The figure also shows the covariance ellipse associated to each corner. Figure 5b shows lines and corners detection. Lines also have their covariance ellipses, but they remain in the (*ρ*, *α*) system. The mobile robot shown in figure 5 is of the unicycle type, whose kinematics were previously shown in (1). The mathematical model associated to corners of the environment is presented in (8), while the mathematical model of a line is shown in (9) [14,15].(8)

In (8) and (9) are shown the observation models used for corners and lines. *ψ*_*R*_, *ψ*_*β*_, *ψ*_*ρ *_and *w*_*α *_are their associated Gaussian noises, and *x*_*v*_(*k*) = [*x*_*v*,*x*_(*k*) *x*_*v*,*y*_(*k*) *x*_*v*,*θ*_(*k*)]^*T *^is the pose of the robot. Figure 5b shows the geometric interpretation of each variable of (8) and (9). More about these features models can be found in [14] and [15].

### SLAM general architecture and consistency analysis

**Figure 6:**
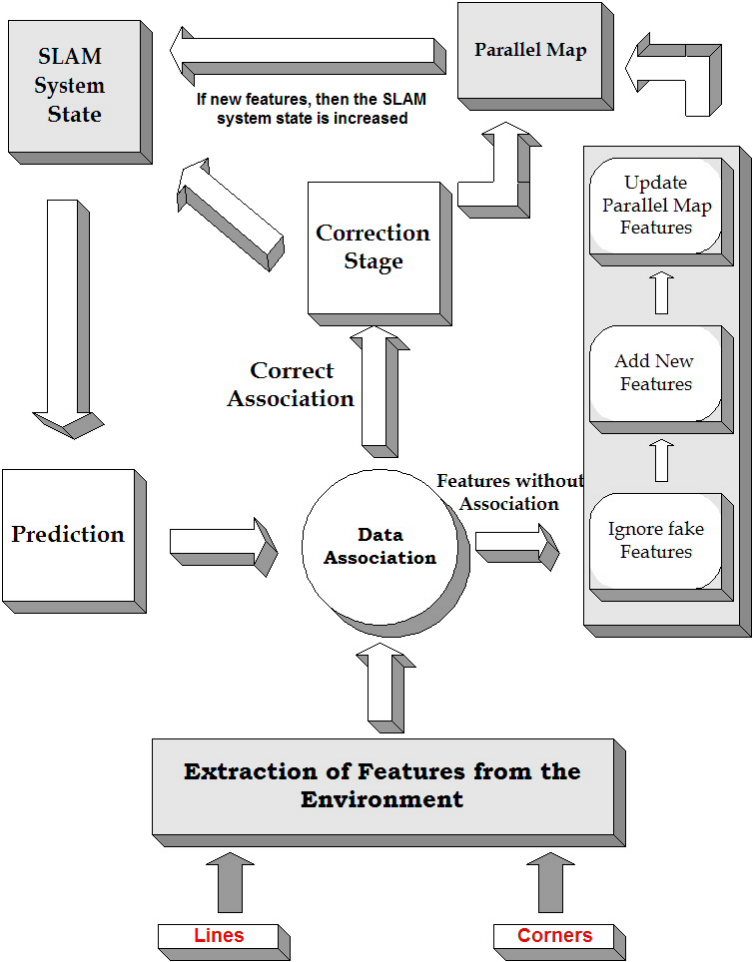
**SLAM general architecture**. Architecture of the EKF-based SLAM algorithm with the parallel map. The parallel map stores the information concerning the beginning and ending points of the walls associated with the detected lines from the environment.

Figure 6 shows the general architecture of the SLAM algorithm implemented in this work. Once the features of the environments are extracted (lines, corners or both) they are compared with the predicted features previously added to the SLAM system state. If there is a correct association between -at least- one feature of the SLAM system state with a recently extracted feature, then the SLAM system state and its covariance matrix are corrected according to the correction stage of the Extended Kalman Filter (see [6]). If there is no appropriate association between the observed features and the predicted ones, the feature is added into the parallel map (if the feature is a line, then their beginning and ending points are incorporated into the parallel map; if it is a corner, then its Cartesian coordinates are added). Once a feature is added into the parallel map, its parameters -according to Eq. (8) or (9)- are initialized in the SLAM system state. When a correction stage is successfully executed, the values stored in the parallel map are also updated according to the changes of their corresponding features in the SLAM system state. The data association criterion used in this work was the Mahalanobis distance [15]. An extended analysis of this SLAM architecture can be found in [36].

**Figure 7:**
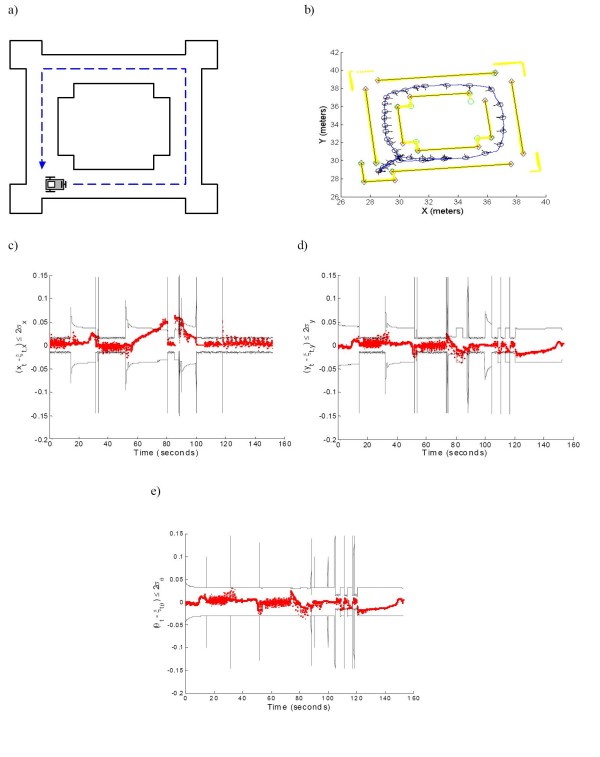
**Consistency test of the EKF-SLAM algorithm**. Consistency test of the EKF-SLAM algorithm used in this paper. a) Simulated environment (solid black lines) with the desired path (dashed blue lines). b) Map reconstruction of the environment. The yellow points correspond to raw laser data; solid black segments represent walls of the environment; green circles correspond to corners; red crosses are the beginning and ending points associated with the segments and the blue line is the path travelled by the mobile robot. c) The error in the *x*--coordinate of the mobile robot is bounded by two times its standard deviation. d) The error in the *y*--coordinate. e) The error in the orientation of the mobile robot.

In addition, figure 7 shows the consistency test of the SLAM algorithm used in this work. The consistency of a SLAM algorithm means that the estimate remains bounded by its standard deviation, ensuring the convergence of the estimation process [7]. In order to get the error of the robot pose, a simulated environment was used. In that way, the true pose of the robot was known at every step of the experiment. Figure 7c-e shows that the error of the robot pose (position and orientation) remains bounded by two times its standard deviation. Figure 7a shows the simulated environment and the robot's initial position. Figure 7b shows the map built by the SLAM algorithm using the information of the parallel map. As it can be seen, the SLAM is consistent.

### Maps managing, visualization and bio-feedback

Once the SLAM algorithm is turned off, the created map is stored and managed by a computer system, [37]. The final map shown to the user is the one with the corners features and segments (the secondary map). Although segments are not part of the system state, lines can not represent the environment to the user, because they may contain several segments and, as was stated, a segment represents a wall of the environment.

The bio-feedback requirements are satisfied since the process works in real time and the user watches the map evolution, his/her biological signals, and the robot's motion in the computer system. More details can be found in [37].

## Results

**Figure 8:**
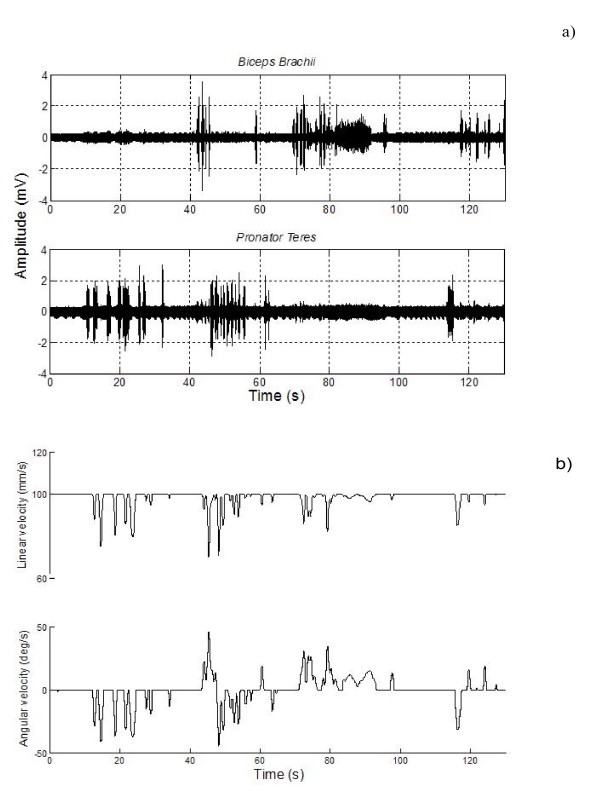
**Muscle signals generated for robot navigation**. Muscle signals generated for robot navigation; a) set of pronation/supination movements to control the robot motion; b) motion command controls.

The previous section introduced the EKF-SLAM used and in the section *Electromyographic Signals Extraction and Classification *the biological signals of the MCI were presented. In this section, the results concerning the implementation of the entire system with the robot navigating in the facilities of the Institute of Automatics of the National University of San Juan, Argentina, are shown. For the experiment shown next, the volunteer has the MCI connected to his/her right arm. The goal is that the mobile robot could close a loop inside the Institute of Automatics. In order to do this, he/she remains in front of the computer system. The signal calibration step was previously made. The signals generated by the user are shown in Figs. 8a-b.

In figure 8a, the *Biceps Brachii *and the *Pronator Teres *signals are the only electromyographic signals shown here. This is so because they are strictly related to the robot motion commands, as it was explained in the section *Electromyographic Signals Extraction and Classification*. Figure 8b shows the motion command control signals obtained from the muscles represented by the signals of figure 8a.

As it was stated in the section *Robotic Devices*, the robot's linear velocity remains fixed (to 100 mm/s) if no angular velocity is presented. Thus, when the robot turns to the right or to the left, the linear velocity decreases. The maximal angular velocity that the robot can reach is 50 deg/s. Both maximal velocity values are appropriate considering that the robot could represent a powered wheelchair. The sampled time in the robotic device is variable, according to the processing SLAM needs.

The mobile robot is connected through Wi-Fi connection with the EMG processing device.

**Figure 9:**
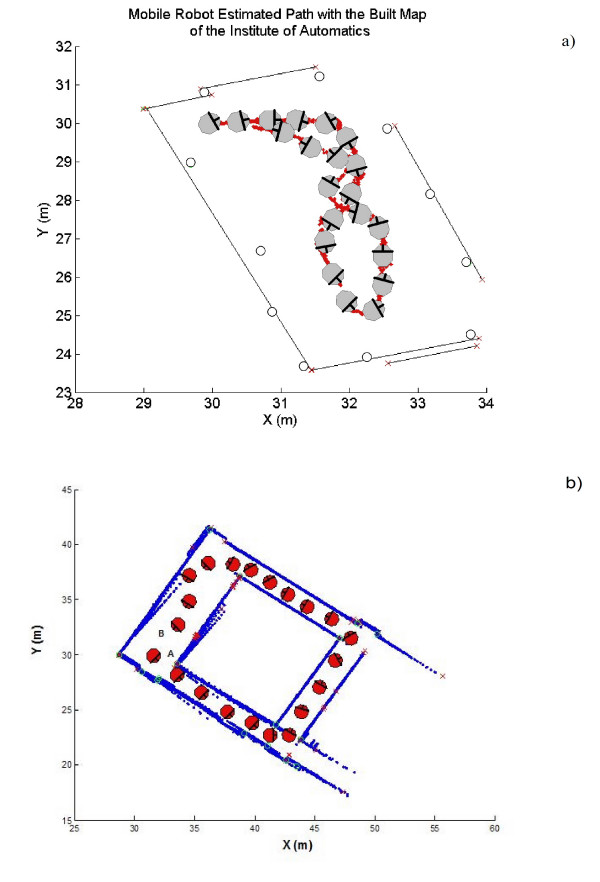
**Maps obtained after navigation**. Maps obtained after navigation; a) Partial construction of the Institute of Automatics using a mobile robot governed by a MCI with SLAM incorporated on it; b) Closing a loop inside the Institute of Automatics, the robot started from point A and traveled around the environment until reaching point B.

Figure 9a shows the partial map of the Institute of Automatics facilities that was built according to the command signals shown in figure 8a-b. The estimated path traveled by the robot is also shown in figure 9a. In figure 9a, segments (representing walls of the environment) are drawn with solid black lines; corners are represented with solid line circles. Figure 9b shows a different experiment. In this case, the robot traveled around the entire Institute. The final map that was obtained is this experiment is shown in figure 9b. Additional files 1 and 2 show a real time experimentation of the system presented in this work.

Closing a loop inside the Institute of Automatic was an experiment that also shown the consistency functionality of the SLAM algorithm. Also, a low level behavioral reactive response allowed the robot to avoid collisions.

### Evaluation parameters and statistical analysis

The seven volunteers were required to repeat the experiment five times, while training and navigation signals were recorded. After completing the trial, each volunteer was asked to fill out a questionnaire including five rating questions to compare their perception of the navigation strategies.

The questionnaire consist of 5 items, which are scored using a 5 point scale of 5 = adequate to 1 = not adequate, and a score of 0 = does not apply. The five items are: (1) personal perception of maneuverability; (2) system response speed; (3) extension of training time; (4) fatigue sensation; (5) facility of use.

**Figure 10:**
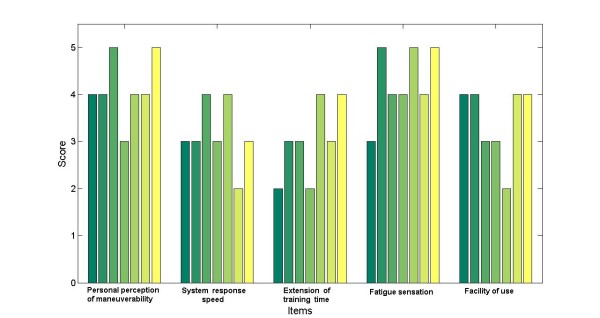
**Statistical analysis**. Subjective rating of the performance of the system based on a questionnaire filled by the volunteers after the trials. The items evaluate maneuverability, response speed, training time, fatigue and how easy is to use. Maximal score is 5. Vertical bars represent the volunteers that took part in the performance evaluation.

All volunteers could successfully complete the navigation tasks and the questionnaire was approved by all users, as figure 10 shows.

The total (training + navigation) time was recorded from each one of the seven volunteers in the five trials. The average total time to complete the loop inside the Institute of Automatics facilities was of 31.8 min, and the particular times are detailed in Table 2. No significant difference in performance was observed between elderly, amputee and young volunteers, showing that this method is adequate to tasks related to navigation and wheeled mobility.

**Table 2:** Average total time of each volunteer

	Mean (min)	Standard Deviation
Volunteer 1	30.60	± 2.8810
Volunteer 2	34.40	± 3.3615
Volunteer 3	29.80	± 2.3875
Volunteer 4	33.80	± 1.6432
Volunteer 5	31.00	± 2.9155
Volunteer 6	30.00	± 2.8284
Volunteer 7	33.00	± 2.5495

## Conclusions

In this paper, a SLAM algorithm applied to a mobile robot governed by a Muscle-Computer Interface (MCI) has been presented. The SLAM algorithm was based on an Extended Kalman Filter and on environmental features extraction. These features are constituted by corners and lines and correspond to walls of the environment and their intersections.

The proposed system improved the autonomy of motor disabled people by the implementation of a general MCI -that could be adapted to the user's capabilities- in order to control the navigation of a mobile robot through unknown environments while mapping them. This mobile robot shares the same kinematics than a powered wheelchair. The SLAM implemented on the vehicle allowed the construction of metric maps. The obtained map was then stored in a computer system for future safe navigation purposes.

The robotic system was managed by a set of basic commands in order to control the movements of the mobile robot. The MCI used in this work was based on the muscles of the right arm of the user although it could be adapted to the patients needs.

The SLAM algorithm in this paper has proven to be a promissory tool for mapping unknown environments which a robotic wheelchair user could travel through. This reduces the need of fixed sensors located in the environment and its a priori knowledge. The user is then able to navigate through environments that he/she is not familiar with. Although the proposed system is for indoors semi-structured environments it could be expanded to outdoors environments in the future. The applications of the system presented in this paper are not restricted to motorized wheelchairs. Once the map of the environment is acquired, the robot is able to drive to any part of the environment where the user sends it and other tasks -as manipulations of objects- can be coupled to the system.

The entire system was tested in a population of seven patients (three elder, two amputees and two young normally limbed patients) showing similar results in all cases and experiments.

## Competing interests

The authors declare that they have no competing interests.

## Authors' contributions

FAC conceived, designed and implemented the SLAM architecture for human-machine interfaces, drafted the document and carried out the experimentations. NL designed and tested the muscle-computer interface and carried out the experimentations. CS implemented the communication system between the SLAM algorithm, the MCI and the mobile robot. FS supervised the project and the MCI performance. FLP supervised the project, drafted the document and participated in the design and coordination of the SLAM experiments. RC supervised the project and the research group, drafted the document and contributed to the discussion of the SLAM and control experimental results. All authors read and approved the final manuscript.

## Supplementary Material

Additional file 1 **Demonstration Video I**. This movie file shows an example of the mobile robot governed by electromyographic signals whereas the vehicle construct a map of the environment using the SLAM algorithm implemented on it. The user of the entire system is an amputee volunteer. Click here for file

Additional file 2 **Demonstration Video II**. This movie file shows another example of the mobile robot governed by electromyographic signals whereas the vehicle navigates inside the Institute of Automatics while constructing a map of the environment using the SLAM algorithm implemented on it. Click here for file
